# Postexposure Treatment and Animal Rabies, Ontario, 1958-2000

**DOI:** 10.3201/eid0802.010177

**Published:** 2002-02

**Authors:** Christopher P. Nunan, Rowland R. Tinline, Janet M. Honig, David G. A. Ball, Peggy Hauschildt, Charles A. LeBer

**Affiliations:** * Ontario Ministry of Natural Resources, Peterborough, Ontario, Canada; † Queen’s University, Kingston, Ontario, Canada; ‡ Ontario Ministry of Health and Long-Term Care, Toronto, Ontario, Canada

**Keywords:** Rabies, Ontario, public health, vaccination, rabies vaccine, treatment

## Abstract

This paper investigates the relationship between animal rabies and postexposure treatment (PET) in Ontario by examining the introduction of human diploid cell vaccine (HDCV) in 1980 and the initiation of an oral rabies vaccination program for wildlife in 1989. Introducing HDCV led to an immediate doubling of treatments. Both animal rabies and human treatments declined rapidly after the vaccination program was introduced, but human treatments have leveled off at approximately 1,000 per year.

Jurisdictions across North America have identified animal rabies as a serious public health concern (*1*,*2*) because the epidemiology of human rabies closely follows the epizoology of animal rabies (*3*). Recent studies have examined the relationship between animal rabies and postexposure treatment (PET) for reasons of surveillance, economic impact, epidemiology, and appropriate treatment (*2*,*4*,*5*). We investigated the nature of this relationship in Ontario from 1958 to 2000, focusing on the impact of two important advances in rabies prevention: a) the introduction of human diploid cell rabies vaccine (HDCV) and b) the initiation of an oral rabies vaccination program (ORVP).

Animal rabies has had a long and varied history in Ontario. Before the 1950s, sporadic outbreaks of rabies occurred, usually associated with dogs. In the early 1950s, a rabies epizootic swept southward from the Arctic, entered northern Ontario in 1954, and by 1958 became enzootic in southern Ontario in red foxes (*Vulpes vulpes*) and striped skunks (*Mephitis mephitis*) (*6*). Sylvatic rabies has, in turn, infected companion animals and livestock, the two groups responsible for most subsequent human exposures (Honig JM, unpub. data, 1985). In 1999, the strain of raccoon rabies that has moved north along the eastern seaboard of the United States entered eastern Ontario from northern New York; by the end of 2000, there were 48 reported cases in raccoons (8 during 1999; 40 during 2000) (*7*). Since 1958, Ontario has averaged 1,200 to 1,300 animal cases per year, for a total of >56,000 cases by 2000. The burden on the public health system has been substantial; for example, >63,000 PETs were reported in the same period. In addition, public health officials have had to investigate all contacts between humans and animals in which rabies may have been transmitted. In the 1980s, for example, at the height of the rabies enzootic, 15,000 to 25,000 such investigations were carried out annually (*8*).

In Canada, all animal rabies collection and laboratory diagnoses are handled by the Canadian Food Inspection Agency (CFIA), the federal ministry responsible for establishing the collection protocols and the laboratory diagnosis of submitted specimens that were suspected of carrying *Rabies virus* (RABV). District veterinary officers throughout the country are responsible for specimen collection and the decision to send specimens to federally operated laboratories for testing.

During the study period, Ontario’s Ministry of Health and Long-Term Care (MOHLTC) distributed vaccine to physicians, free of charge, for the prevention of human rabies. In the fiscal year 1980-81, the ministry began distributing the newly licensed HDCV to replace earlier Semple and duck embryo vaccines. By the fiscal year 1983-84, all distributed vaccines were HDCV (*9*). HDCV was an important advance in rabies prevention because “it is a better immunogen with fewer side effects and requires far fewer doses than the previously recommended duck embryo vaccines” (*10*).

A second important advance in rabies prevention was ORVP. In 1989, the Ontario Ministry of Natural Resources initiated an ORVP in eastern Ontario, targeting the principal wildlife vectors (*11*). By 1994, the ministry had extended ORVP to cover the epizootic area in southern Ontario, and over 1 million vaccine baits were dropped annually. The program resulted in a dramatic drop in rabies incidence in southern Ontario (*11*).

## Methods

We gathered the PET and animal rabies data (Table) from two government agencies. The MOHLTC annual reports from 1958 to 1978 list the number of courses of rabies vaccine distributed in each calendar year; for this paper, we considered each such course as a PET. For 1979 to 1988 and 1998 to 2000, we obtained similar records directly from internal reports in the MOHLTC. For 1989 to 1997, we obtained vaccine distribution data from the Public Health and Epidemiology Reports for Ontario (*12*). We obtained the annual number of laboratory-confirmed cases of animal rabies in Ontario directly from CFIA. Data were compiled on all terrestrial animals and bats that tested positive for RABV. Data on the number of negative test results were not available.

**Table:** Rabies postexposure treatment (PET) and laboratory-confirmed animal rabies, Ontario, 1958 to 2000

Year	PET	Animal rabies	Ratio PET to animals rabies	PET rate per 100,000^a^
1958	1,647	2,426	0.7	28.3
1959	479	1,210	0.4	8.0
1960	566	241	2.3	9.3
1961	790	636	1.2	12.7
1962	991	879	1.1	15.6
1963	965	907	1.1	14.9
1964	852	1,006	0.8	12.9
1965	1,367	1,352	1.0	20.2
1966	1,168	1,004	1.2	16.8
1967	1,461	1,232	1.2	20.5
1968	1,539	1,924	0.8	21.2
1969	1,187	2,154	0.6	16.1
1970	1,164	1,477	0.8	15.4
1971	960	1,428	0.7	12.4
1972	1,252	2,161	0.6	15.8
1973	1,020	1,503	0.7	12.7
1974	974	1,425	0.7	11.9
1975	1,050	1,954	0.5	12.6
1976	935	1,395	0.7	11.1
1977	957	1,267	0.8	11.3
1978	816	1,422	0.6	9.5
1979	1,002	1,480	0.7	12.0
1980	1,096	1,412	0.8	11.6
Average	1,054	1,387	0.8	14.3
1981	1,833	1,333	1.4	20.8
1982	2,402	2,095	1.1	27.0
1983	2,481	1,834	1.4	27.5
1984	2,027	1,366	1.5	22.1
1985	2,150	1,975	1.1	23.2
1986	4,212	3,274	1.3	44.7
1987	2,621	2,001	1.3	27.2
1988	2,266	1,830	1.2	23.1
1989	2,640	1,870	1.4	26.2
1990	1,991	1,611	1.2	19.4
1991	1,739	1,234	1.4	16.7
1992	2,186	1,371	1.6	20.7
1993	2,581	1,241	2.1	24.2
1994	1,437	613	2.3	13.3
1995	1,182	328	3.6	10.8
1996	937	149	6.3	8.5
1997	1,079	95	11.4	9.6
1998	1,048	80	13.1	9.2
1999	890	100	8.9	7.7
2000	1,073	183	5.9	9.2
Average	1,939	1,229	1.6	19.0

^a^Population figures are from Statistics Canada Quarterly Estimates of Population For Canada, Provinces and Territories, 1951-2000.

Our PET and rabies data were maintained for the entire study period by two central government agencies with a consistent mandate for collecting and reporting. Unfortunately, because these two agencies operate independently, we could not match the individual human treatments to the specific specimens that tested positive for rabies.

Human population data were obtained from Statistics Canada Quarterly Estimates of Population for Canada, Provinces and Territories, 1951-2000.

We used regression analysis to examine the relationship between PET and the number of laboratory-confirmed cases of rabies in terrestrial animals and bats in Ontario. Analyses were done for the periods 1958 to 1980 and 1981 to 2000. As previously noted, HDCV was used during the second period. We used SPSS (release 10.0.5, SPSS Inc., Chicago, IL**)** to perform the regressions.

## Results

From 1958 to 1980, the ratio of human treatments to animal cases was <1 in most years (Table, Figure). After HDCV was introduced in 1980, the yearly ratios of human treatments to animal cases were >1. Furthermore, from 1980 to 1981, the rate of PET per 100,000 persons almost doubled. The annual number of PETs increased from an average of approximately 1,000 in the 1970s to an average of more than 2,000 per year during the 1980s. During the 1980s and early 1990s, the annual number of PETs closely paralleled the annual number of animal cases.

The regression for the 1958 to 1980 period showed a weak but significant relationship between PET and animal rabies (R^2^=0.42, p<0.001, n = 23, intercept = 557 [standard error, SE, 135.4], slope = 0.358 [SE 0.092]). After 1980 the relationship was much stronger (R^2^=0.91, p<0.001, n = 20, intercept = 861 [SE 100.5], slope = 0.877 [SE 0.067]). The slopes of these regressions indicate that before 1980, there were approximately three reports of rabid animals for every PET, whereas after 1980, the ratio was approximately 1:1. Finally, the regression demonstrates that the base level of treatments after 1980 was 861, approximately 55% higher than the base level (557) before 1980.

Following the initiation of ORVP, the regular cycle of animal rabies was broken in the early 1990s (Figure) and the number of laboratory-confirmed rabid animals declined. The number of human treatments also declined by 50%, from more than 2,000 per year throughout most of the 1980s to approximately 1,000 per year in the late 1990s.

**Figure:**
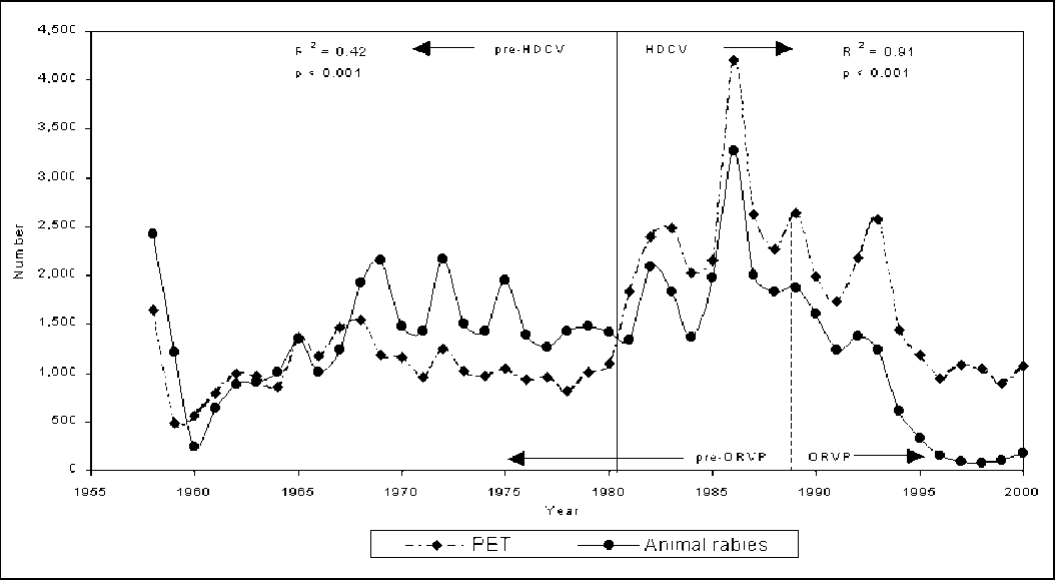
Postexposure treatment (PET) and laboratory-confirmed animal rabies. HCRV, human diploid cell vaccine; ORVP, oral rabies vaccination program; R^2^**,** coefficient of determination.

During the period 1968 to 1980, there was an apparent change in the relationship between PET and animal rabies compared with the initial 1958 to 1967 period of the enzootic (Figure, Table). The ratios of PET per rabid animal and PET rate per 100,000 persons for the 1958 to 1967 period were 0.9 and 16.0, respectively, and declined to mean values of 0.7 and 13.2, respectively, for the 1968 to 1980 period.

## Discussion

There was a dramatic change in the relationship between PET and animal rabies coincident with the introduction of HDCV in 1980. Furthermore, after 1980, the positive correlation between human treatments and animal rabies strengthened. We could not find any directives from the MOHLTC or published studies indicating a change in treatment policy when HDCV was introduced. Since HDCV had fewer side effects and the vaccination regime was simpler and less traumatic than previous treatments, there was, perhaps, less reluctance to administer PET after 1980 and, therefore, the use of PET increased and paralleled the incidence of animal rabies more closely.

When Ontario began its ORVP, one of the arguments for it was that animal rabies would be reduced and human treatments would follow suit. Our findings support this argument and are consistent with reports from other jurisdictions (*3*,*13*,*14*). The decline in human treatments in Ontario, however, was not as rapid as the decline in rabies cases. In fact, in recent years, while animal rabies incidence dropped to approximately 100 to 200 reported cases per year, PET leveled off at approximately 1,000 per year, about the same level as immediately before HDCV was introduced. We suspect the reasons for the continued high ratio of PET to animal rabies are varied and complex. For instance, with the continued presence of rabies, if a suspect animal is not available for testing, PET is administered as a precautionary measure. Furthermore, rabies in bats has been unaffected by the ORVP. Bats have been implicated in more than half (15 of 28 cases) of the human rabies cases diagnosed in the United States since 1980 (*15*) and in a recent death in Quebec (*16*). The recent spread of raccoon rabies from New York State into southeastern Ontario has increased the media coverage of rabies and will contribute to uncertainty about the presence of the RABV in the province. Under these circumstances, with the relatively safe HDCV, a continuing high number of treatments should be expected. Even in the absence of further rabies cases, the regression results suggest, at the 95% confidence interval, annual treatments would range from 650 to 1,072 annually.

The decline in PET per rabid animal and PET rate per 100,000 persons in the 1968 to 1980 period hints at other factors affecting the relationship between animal rabies and the administration of PET. We were unable to find any evidence in published studies detailing a change of government policy about the administration of PET or some traumatic event that could initiate a de facto policy change. Indeed, studies during that period recommended a treatment approach similar to today’s guidelines (*17*). Furthermore, compulsory vaccination of companion animals was not an explanation. Under the Health Protection and Promotion Act, a regulation governing rabies immunization was not introduced until 1984 and it has taken until 2000 for all district health units in southern Ontario to be included in it (*18*). All we know is that, early in this period, rabies incidence across southern Ontario had stabilized and developed regular cycles in various regions (*19*). We can only speculate that, as animal rabies incidence became more predictable, health professionals and the public learned to manage the risk and there was less pressure to give PET, especially with older vaccines and their lengthy regime of injections. Experience in the United States indicated that consultation with state health departments during management of potential rabies exposure reduced PETs (*10*).

##  Conclusions

Our data suggest that human interventions have played a major role in the relationship between PET and animal rabies. The introduction of a new, safer vaccine was associated with a sudden increase in the number of PETs per rabid animal. Furthermore, while the introduction of an ORVP reduced animal rabies, PET did not drop at a similar rate and has appeared to stabilize at approximately 1,000 persons per year. This stabilization, despite the diminishing number of rabies cases, is important in estimating the economic impact of rabies control and public education. However, as our data for the 1968 to 1980 period show, there are other, as-yet-unknown factors that affect the animal rabies/PET relationship.

We believe that two general approaches are needed for the future study of this complex relationship. First, we need details of the circumstances of rabies incidents involving human exposures, such as those assembled by Moran et al. (*5*), Honig (unpub. data), and the Public Health Branch, Ministry of Health (*12*). For Ontario, assembling these data will require follow-up interviews on a case-by-case basis. Second, if we can obtain data on the distribution of PETs by the 32 health units in southern Ontario (we have distribution data for animal rabies), we may gain further insight by (a) examining the distribution and interaction of human and animal populations; (b) investigating the influence of the geographic scale at which the relationships are examined; and (c) making regional comparisons of the administration of PET and the relative surveillance efforts in an area over time, given the history of rabies incidence and public awareness campaigns in the area.
